# PTP1B regulates lymphocytes responses androgen deprivation

**DOI:** 10.1186/2051-1426-2-S1-P2

**Published:** 2014-02-24

**Authors:** Simo Arredouani, Haydn Kissick, Laura Dunn, Martin Sanda

**Affiliations:** 1 Department of Surgery, Harvard Medical School, Boston, MA, USA; 2 Emory University School of Medicine, Atlanta, GA, USA

## 

Androgen deprivation therapy (ADT) synergizes with prostate cancer (PCa) immunotherapy through androgen receptor- (AR) mediated immunoregulation. We show that surgical castration of male mice induces dramatic alterations in CD4+ T cell transcriptome. Pathway analysis identified T Cell Differentiation as the most altered pathway. Consistently, a significant increase in expression of T-bet and IFN-γ was observed, whereas Gata-3, Foxp3, and Ror-γt were not affected, suggesting a preferential differentiation of CD4 T cells into the Th1 phenotype. Transcription factor profiling showed a significant activation of the Th1-related factors IRF-1, -3 and -7, and STAT1. Castration reduced the threshold of responsiveness to IL-12, resulting in enhanced T-bet expression and STAT4 phosphorylation. In vitro and in vivo tests suggested an inducing effect of testosterone on the expression of EGR2 and PTP1B, two major players in tolerance and anergy. PTP1B was also elevated in CD4+ T cells from PBMC of PCa patients on ADT. More interestingly, we showed that AR binds to the PTP1B gene, whereas no binding was shown on EGR2 gene, suggesting a differential effect on PTP1B and EGR2. Finally, we demonstrated that inhibition of PTP1B restored Tyk2 phosphorylation. Together, our findings establish PTP1B and EGR2 as rational targets for immunotherapy of PCa.

